# Radial nerve palsy after the use of an adjuvant hinged external fixator in a complex fracture–dislocation of the elbow: a case report and review of the literature

**DOI:** 10.1186/s13256-016-0904-9

**Published:** 2016-05-11

**Authors:** Pietro Poglia, Laurent Wehrli, Sylvain Steinmetz, Philippe Zermatten

**Affiliations:** 1 Department of Surgery, Division of Orthopaedic Surgery and Traumatology, Centre Hospitalier du Centre du Valais, avenue Gd-Champsec 80, 1950 Sion, Switzerland; 2 Department of Surgery, Division of Plastic and Hand Surgery, Centre Hospitalier Universitaire Vaudois, University of Lausanne, rue du Bugnon 46, 1011 Lausanne, Switzerland

**Keywords:** Elbow, Fracture–dislocation, Technique, Morbidity

## Abstract

**Background:**

The combination of an elbow dislocation, a radial head fracture, and a coronoid process fracture is known as “terrible triad” injury of the elbow. This injury is one of the most challenging injuries of the musculoskeletal system and almost always causes instability of the elbow. The use of an adjuvant hinged external fixator in such injuries is still debated.

**Case presentation:**

In this case report we present a case of radial nerve palsy after setting up an adjuvant hinged external fixator in a complex fracture–dislocation of the elbow. The patient was a 39-year-old white man. A revision of his radial nerve was undertaken at 7 weeks. A radial nerve injury at two levels facing the humeral apex pins was found intraoperatively; the pins were carefully removed and partial nerve grafts done. The functional outcome at 18 months was excellent.

**Conclusion:**

This case report highlights that the use of an adjuvant hinged external fixator in complex fracture –dislocation of the elbow is technically demanding and not without risk.

## Background

Fracture–dislocations of the elbow remain one of the most difficult injuries to manage in traumatology. A complex elbow dislocation combined with radial head and coronoid process fractures was named the “terrible triad” by Hotchkiss [1] because of historically poor outcomes. Most patients presenting with this type of injury require surgery. The principles of surgical management are based on the understanding of elbow pathomechanics [2–9]. Once the primary and secondary stabilizers of the elbow [10] have been fixed and reconstructed an adjuvant hinged external fixator can be used to protect the healing ligaments and allow an early mobilization [11–15]. The use of such a device is technically demanding and not totally without risk. We report on a case of radial nerve palsy after the use of an adjuvant hinged external fixator in a complex fracture–dislocation of the elbow. This complication is well known. However, if anatomical and technical considerations are respected, the patient could be spared this inconvenience. Based on our case and a literature review, we discuss the management of the “terrible triad” injury of the elbow.

## Case presentation

**Fig. 1:**
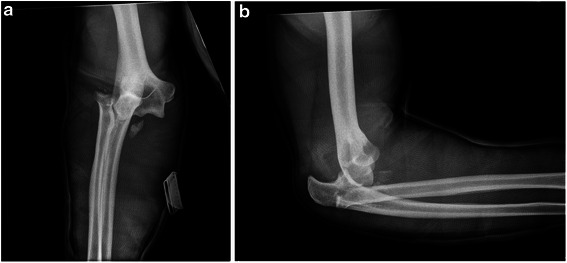
Anteroposterior (**a**) and lateral (**b**) views of the right elbow show a fracture–dislocation

**Fig. 2:**
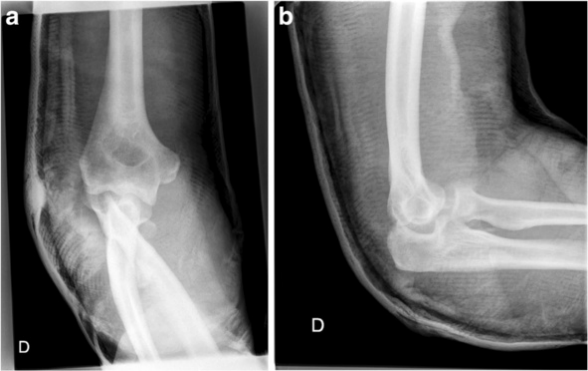
Postreduction radiographs (anteroposterior (**a**) and lateral (**b**) views) show an ulnohumeral joint partially reduced, a comminuted radial head fracture and a fracture of the coronoid process

**Fig. 3:**
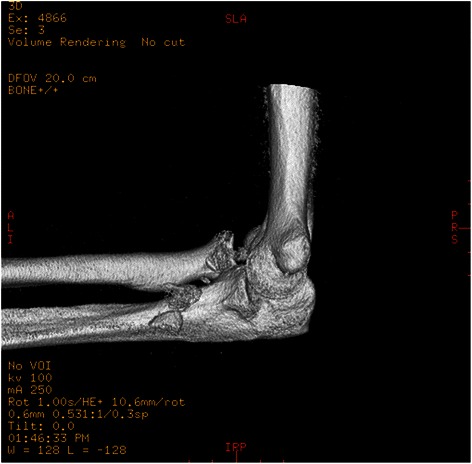
Computed tomography (three-dimensional reconstruction)

**Fig. 4:**
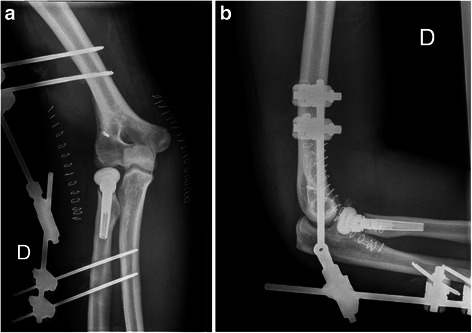
Postoperative radiographs: anteroposterior (**a**) and lateral (**b**) views. The radiographs show a radial head prosthesis *in situ*, indirect signs of collateral ligamentous repair (anchor), and a congruency of the ulnohumeral joint

A 39-year-old white man fell from a height of 3 meters landing on his right dominant arm in extension. He initially presented to his family physician who made the radiological diagnosis of a fracture–dislocation of the right elbow (Fig. 1a, b), applied a splint and sent him to our hospital where he arrived approximatively 5 hours after the time of injury. In the emergency room we began under general anesthesia a reduction and immobilization of his elbow in a splint including his wrist with his forearm in pronation and his elbow in flexion because of a major instability. The postreduction radiographs (Fig. 2a, b) showed an ulnohumeral joint partially reduced, a comminuted radial head fracture (type Mason III) [16], a fracture of his coronoid process (type Regan–Morrey I) [17], and indirect signs of collateral ligamentous injuries. There was no associated vascular injury. Three-dimensional computed tomography showed more precisely the abovementioned lesions (Fig. 3). The definitive surgical treatment was planned 5 days later. His radial head was replaced by an anatomical prosthesis (MoPyC, BioProfile® by Tornier), the lateral collateral ligament was refixed to his epicondyle by means of an anchor (GII™ Anchor, DePuy Mitek), the anterior fascicle of his medial collateral ligament was sutured, his partially torn flexor-pronator mass was repaired by reabsorbable sutures, and an adjuvant hinged external fixator (DJDII™, Stryker) was placed to protect the reconstruction of his capsuloligamentous structures and allow an early mobilization of his elbow. Once the rotational axis of his elbow was determined by means of a humeral viewfinder, apex pins were introduced into his distal humerus (two pins of 4-mm diameter) and his proximal ulna (two pins of 3-mm diameter) using the guides through mini-incisions and their position was controlled under fluoroscopy. The external unilateral assembly was then completed with the couplings and rods (Fig. 4a, b). In the recovery room we observed complete radial nerve palsy with a fall-hand and paresthesia/hypesthesia facing the “snuff box” and the dorsal part of his thumb. A static splint was applied during the night and ergotherapy with dynamic orthosis was prescribed.

**Fig. 5:**
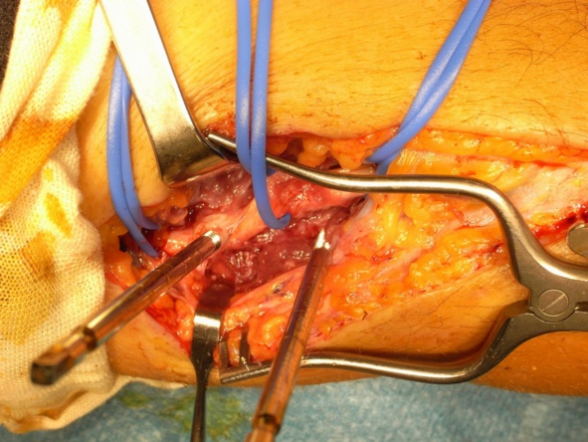
Perioperative picture showing a penetrating lesion of the radial nerve caused by the proximal pin and a lesion of the posterior margin of the same nerve more distally (caused by the distal pin)

**Fig. 6:**
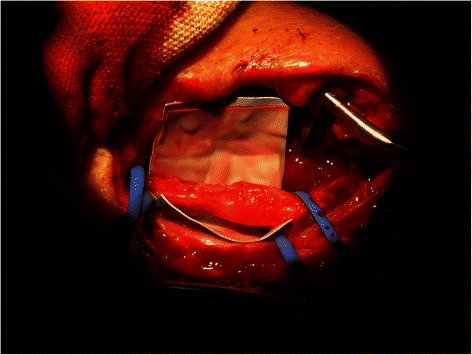
Perioperative picture showing partial nerve grafts of the radial nerve

Electroneuromyography (ENMG) at 2 weeks postoperatively showed a severe injury of his radial nerve. This examination could not differentiate at that time between an axonotmesis and a neurotmesis. An ENMG at 5 weeks postoperatively enabled us to differentiate and we concluded that his radial nerve injury was an axonotmesis. A revision of his radial nerve was undertaken at 7 weeks postoperatively. The surgeon (L.W.) described perioperatively a radial nerve injury at two levels facing the humeral apex pins. These were carefully removed. The proximal pin had caused a penetrating lesion and the distal pin a lesion of the posterior margin of the radial nerve (Fig. 5). He began partial nerve grafts (Fig. 6). He took one of the three branches of the sensitive branch of the radial nerve at the level of the patient’s elbow.

**Fig. 7:**
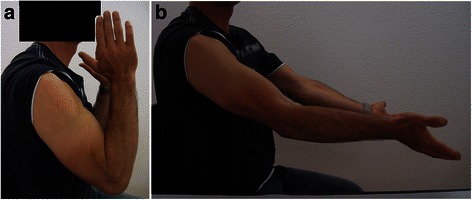
Range of motion of the right elbow at 18 months: flexion (**a**) and extension (**b**)

Early mobilization of the patient’s elbow was prescribed and a static splint in extension applied only at night for 1 week. The patient was reviewed at 3, 6, 12, and 18 months. At the last follow-up he reported residual paresthesia facing the first dorsal web space of his right hand. A clinical examination showed an arc of motion greater than 100 degrees (Fig. 7a, b), excellent stability of his elbow, sensitivity in the radial area of his arm practically symmetric, and a grip strength of 27 kg compared to 41 kg on the opposite side. His Mayo Elbow Performance Score (MEPS) was 180. He returned to work at full capacity as person in charge of a cleaning agency.

## Discussion

A clear understanding of elbow pathomechanics [2–9] is essential to properly treat these complex fracture–dislocations. The major determinant of stability of the elbow is the ulnohumeral joint, particularly the coronoid process. An *et al.* [18] demonstrated experimentally that the relative contribution of the olecranon in resisting various loading configurations was linearly correlated with the extent of resection of the proximal part of the ulna. Based on clinical experience the critical amount of ulnohumeral joint required for maintaining stability is approximately 50 % of the olecranon. The contribution of the radiohumeral joint to elbow stability is intimately related to, and dependent on, the integrity of the collateral ligaments. The major structure resisting initial valgus displacement, even with an intact radial head, is the medial collateral ligament. If this ligament is attenuated or torn, the radial head assumes the role of a secondary stabilizer. Laterally the ligamentous complex originally described by Martin [19] and further refined by O’Driscoll *et al*. [20–22] is relevant to a condition termed “posterolateral rotatory instability” (PLRI). PLRI is nowadays well recognized to be associated with lateral ulnar collateral deficiency and most often occurs with fracture–dislocations of the elbow. The role of the coronoid process contributing to stability is complicated by defining its relevance with or without the radial head. At least 50 % of the coronoid must be present for the ulnohumeral joint to function. Absence of the radial head further and dramatically compromises the elbow with a 50 % coronoid deficiency.

The terrible triad injury is often caused by a fall on an outstretched hand as in our case. A posteriorly directed force results from a fall on an extended elbow which levers the ulna out of the trochlea. O’Driscoll *et al*. [23] described an additional valgus stress and/or posterolateral “roll out” that occurs with this injury. A clinical examination should note any signs or symptoms of neurovascular injury and skin or soft tissue compromise. When preliminary radiographs confirm the presence of an elbow fracture–dislocation initial management begins with a closed reduction under conscious sedation administered intravenously or general anesthesia. After reduction is achieved the elbow should be brought through the range of motion (ROM) to test stability in all planes with the forearm in pronation, neutral, and supination. A second neurovascular status has to be done and any change should be noted. Prereduction and postreduction imaging includes anteroposterior and lateral radiographs. Computed tomography is also routinely used.

Most patients presenting a terrible triad injury of the elbow require surgery for stabilization. The first principle for treating this complex injury is to restore the ulnohumeral joint. This is done by reduction of the joint and fixation of the olecranon and/or the coronoid process. The second principle is that the radial head is an important stabilizer which must be fixed or replaced if the ulnohumeral joint has been compromised. The lateral collateral ligaments should be repaired in all cases. If the medial collateral ligament is deficient it is repaired or the elbow could be stabilized by a hinged external fixator. In our case we replaced the radial head by an anatomical prosthesis because of its comminution, reattached the lateral collateral ligament to the epicondyle by means of an anchor, reconstructed the anterior fascicle of the medial collateral ligament, repaired the partially torn flexor pronator mass by reabsorbable sutures, and placed a hinged external fixator to protect the ligamentous reconstruction and allow an early mobilization.

The results of elbow dislocations with associated radial head and coronoid fractures are historically poor because of recurrent instability and stiffness from prolonged immobilization. Nowadays, many authors [24–29] have managed these injuries with a standard surgical protocol postulating that early intervention, stable fixation, and repair of associated capsular and lateral ligamentous injuries and, in selected cases, repair of the medial collateral ligament and/or adjuvant hinged external fixation would provide sufficient stability to allow motion at 7 to 10 days postoperatively and enhance functional outcome. The use of an adjuvant hinged external fixator of the elbow is technically demanding and requires accurate alignment of the fixator axis with the anatomic axis of the elbow. Common complications include pin loosening, pin tract infection or fracture, injury to adjacent neurovascular structures, and loss of reduction [14, 30–32]. In our case, we described an atypical radial nerve injury at two levels due to the improper placement of the humeral apex pins. To avoid such a complication we think that it is very important to check the correct placement of the humeral apex pins before their insertion either under fluoroscopic control or through a mini-open approach to visualize the radial nerve.

## Conclusions

Long-term outcome with surgical management of complex elbow fracture–dislocations is as yet unknown. The use of an adjuvant hinged external fixator is technically demanding and not without risk, which is why its indication should be limited to selected cases.

## Consent

Written informed consent was obtained from the patient for publication of this case report and any accompanying images. A copy of the written consent is available for review by the Editor-in-Chief of this journal.
